# Antibacterial activity of different root canal sealers against *Enterococcus faecalis*

**DOI:** 10.4317/jced.53753

**Published:** 2017-06-01

**Authors:** Claudio Poggio, Federico Trovati, Matteo Ceci, Marco Colombo, Giampiero Pietrocola

**Affiliations:** 1 Department of Clinical-Surgical, Diagnostic and Pediatric Sciences – Section of Dentistry, University of Pavia, Pavia, Italy; 2 Department of Molecular Medicine – Unit of Biochemistry, University of Pavia, Pavia, Italy

## Abstract

**Background:**

The aim of the present study was to compare in vitro the antimicrobial activity of different root canal sealers against *Enterococcus faecalis*, prior and subsequent to setting.

**Material and Methods:**

Agar diffusion test (ADT) was used for evaluating the antibacterial activity of non-set sealer while the direct contact test (DCT) was used for after setting.

**Results:**

ADT: Except for TotalFill BC Sealer all the others sealers tested showed antibacterial activity. BioRoot™RCS, MTA Fillapex and Sealapex Root Canal Sealer showed the lowest antibacterial activity, a significant increase in antibacterial effect for both Pulp Canal Sealer™ and AH plus sealers were found. Significantly higher were the mean diameters of the bacterial inhibition zone by both EasySeal or N2 sealers. DCT: AH plus and Sealapex Root Canal Sealer doesn’t show any bactericidal effect after 6 min of contact. After 15 and 60 min of contact a significant increment for AH plus and for Sealapex Root Canal Sealer of the bactericidal effect was found. Significantly much higher was the antibacterial effect of Sealapex Root Canal Sealer compare to that observed for AH plus. BioRootTMRCS, MTA Fillapex, Pulp Canal Sealer™ and N2 showed at least means of the number of colonies formed in milliliter after 6 min of contact. Except for N2, a significant increase in bactericidal effect after 15 and 60 min for the other compared sealers (BioRootTMRCS, MTA Fillapex and Pulp Canal Sealer™).

**Conclusions:**

For every contact times considered, both TotalFill BC Sealer and EasySeal were bactericidal against *E. faecalis* and killed all bacteria.

** Key words:**Agar diffusion test, antibacterial activity, direct contact test, Enterococcus faecalis, root canal sealer.

## Introduction

Endodontic failures may be explained by a myriad of different factors. The persistence of intraradicular or secondary infections is the main causes of failed root canal treatment (1). Even if the eradication of microorganism from the root canal space, or at least their reduction to levels compatible with periradicular tissue health are the objectives of endodontic treatment; many studies reported the presence of bacteria in dentinal tubules and cementum even after treatment (2).

The persistence of microorganisms may be due to ineffective intracanal irrigation, mechanical preparation that leaves much of the root canal surfaces untouched and ineffective chemo-mechanical preparation due to anatomical limitations (3).

It has been demonstrated that microorganisms of teeth with failed endodontic treatment significantly differ from that normally found in untreated teeth (4). *Enterococcus faecalis*, facultative Gram-positive cocci, is present in over one third of the canals of teeth with persisting periapical lesions (5).

Since complete eradication of microorganism from the endodontic space is not predictable; the antimicrobial activity of root canal sealers may help to eliminate residual microorganisms unaffected by chemomechanical preparation of the root canal system (6). Therefore, endodontic sealers with high antimicrobial activity helps to decrease or prevent the growth of microorganisms and aid the repair process of apical and periapical tissues (7).

Commercial sealers presently available differ in their chemical composition as well as in their physico-chemical properties; sealing ability, biocompatibility, adhesiveness, insolubility to oral and tissue fluids, and dimensional stability (8). Eugenol containing sealers are known for their antibacterial effect, which was higher against *E. faecalis* than calcium hydroxide-based sealers and resin-based sealers (9).

Many studies have investigated the antibacterial effect of endodontic sealers. The agar diffusion test (ADT) is one of the most commonly used techniques to evaluate dental materials. ADT was used for evaluating the antibacterial activity of non-set sealer while the direct contact test (DCT) was used for after setting. Therefore, the aim of the present study was to compare in vitro, by both ADT and DCT the antimicrobial activity of different root canal sealers against *E. faecalis*, prior and subsequent to setting.

## Material and Methods

Eigth root canal sealers were tested: BioRoot™RCS, TotalFill BC Sealer, MTA Fillapex, Sealapex Root Canal Sealer, AH Plus, EasySeal, Pulp Canal Sealer™, N2.  Table 1 shows chemical composition and characteristics of the tested sealers.

**Table 1:**
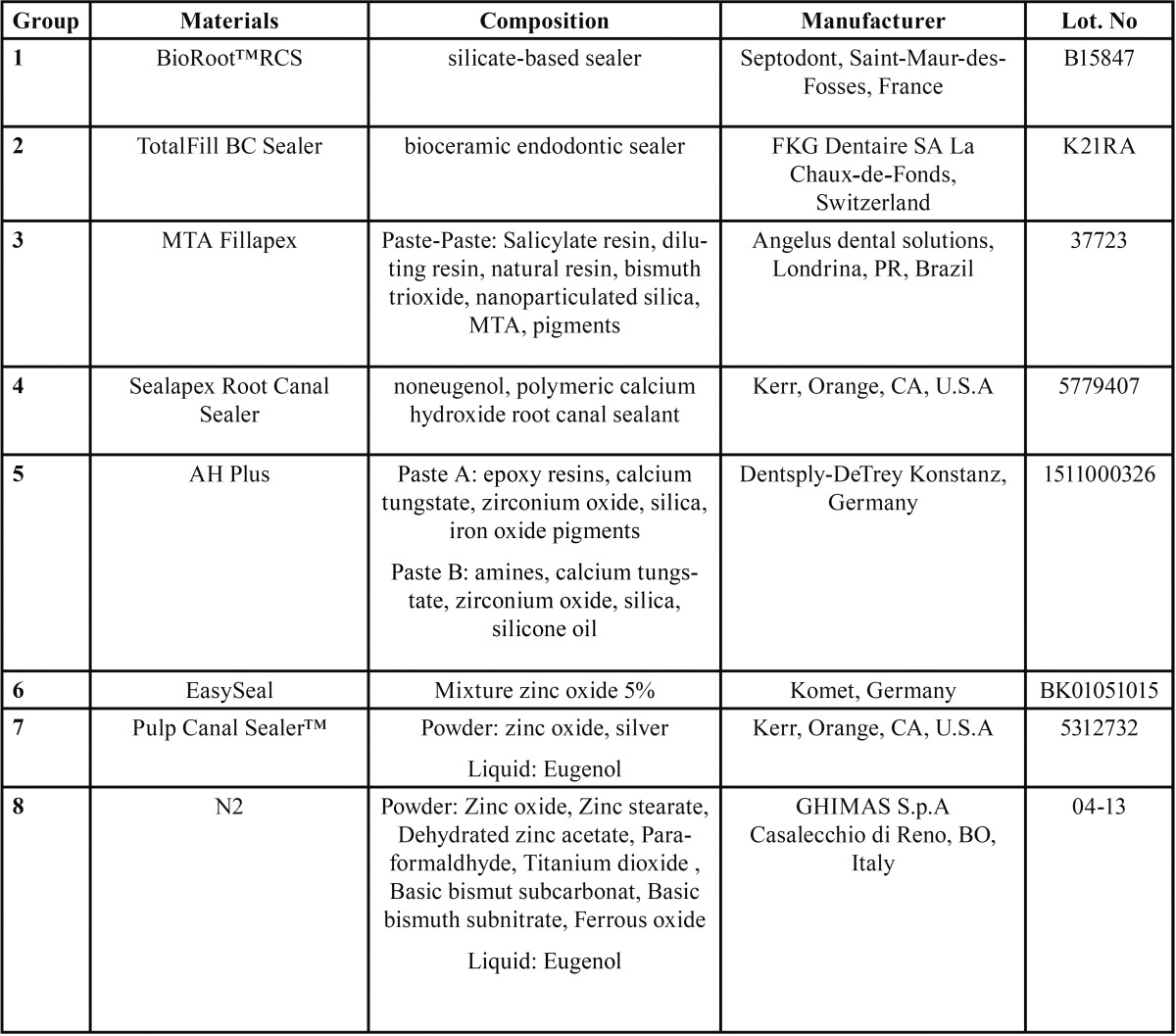
Composition, manufacturer and lot number of tested materials.

Agar diffusion test (ADT)

The microbiological assays were carried out under aseptic conditions in a laminar flow chamber (Foster Wheeler Italiana S.p.A, VBH C2 biohazard cabine). The antibacterial activity was evaluated using a standard strain of *Enterococcus faecalis* (ATCC 29212). The microorganisms were cultivated in Brain Heart Infusion – BHI broth (Merck, Darmstadt, Germany) at 37°C for 18 h. Then, a bacterial suspension was prepared with 0.85% saline solution to match the turbidity equivalent to 0.5 McFarland standard tube, corresponding to 1,5 x 108 CFU mL-1. Six replica plates containing Brain Heart Infusion agar (Difco Lab., Detroit, MI, USA) were spread with 0.1 mL of the bacterial suspension, using a Drigalsky’s loop. Thereafter, four wells of 6 mm in diameter and 4 mm in depth (one for each material) were made with a punch by removing the agar at equidistant points and then filled immediately with the materials to be evaluated. Two plates did not receive the bacterial suspension; one did not receive the sealers and aimed to control the sterilization of the culture medium, whilst the other received the sealers and aimed to control their contamination. All plates were maintained at room temperature for 2 h for prediffusion of the materials and then incubated at 37ºC for 48 h under aerobic conditions. The inhibition zones around each one of the wells were then measured by the same operator in two perpendicular locations with a millimeter ruler (sliding calipers) with accuracy of 0.5 mm. The size of the inhibition zone was calculated as follows: size of inhibition zone = (diameter of halo – diameter of specimen) x ½ All the assays were conducted in triplicate and the results were recorded in terms of the average diameter of inhibition zone.

Direct contact test (DCT)

The DCT was used to evaluate the antibacterial properties of the root canal sealers by counting the number of bacterial colonies after plating on agar plates. All sealers were mixed based on manufacturer’s instructions and were place in sterile cylinder-shaped plastic blocks with diameter of 5 mm and the depth of 5 mm. The samples were placed in an incubator at 37°C and the humidity of 100% for a period of 7 days. The obtained sealer blocks were grinded and powdered using a ceramic mixer (Coors Tek, Goled Co, USA). The powder were placed in special sterile packs and sterilized with ethylene oxide gas. Fifty milligrams of each sealers powder was weighed by using a precision balance (Mettler-Toledo, model AE1633, Novate Milanese, Italy, metering accuracy 0.01 mg) and 1 ml of sterilized saline suspension was added to each powdered sealer using sterile pipettes so that a suspension with the density of 50 mg/ml produced. The bacterial suspension with standard density of 0.5 McFarland (1,5 x 108/ml) were prepared. Equal volumes of bacterial suspension and the sealer suspension (1ml) were mixed using a Bench Mixer Vortexer (Sigma, St. Louis, MO, USA). The sealer-free saline suspension was considered as the positive control. Six, fifteen and sixty minutes after mixing, the suspensions were diluted ten thousand times, and 0.01 ml of the diluted suspension was plated in triplicate on the already-provided BHI agar plates (Difco Lab., Detroit, MI, USA). After incubation at 37°C for 24 h, the colonies formed on the agar plated were counted. Then, the number of colony-forming unit (CFU) was calculated for each sealer for the different times of the experiment. These experiments were repeated three times.

Statistical methods

Statistical analysis was carried out using GraphPad Prism statistical analysis software. Differences between groups were analyzed by Student’s t test. Two-tailed *P* values of 0.05 were considered statistically significant.

## Results

In this *in vitro* study, the antibacterial effect of eight root canal sealers on *Enterococcus faecalis* strain was tested. For this purpose, both agar diffusion test on freshly mixed sealers or direct contact test on set sealers were employed.

Agar diffusion test (ADT)

**Figure 1:**
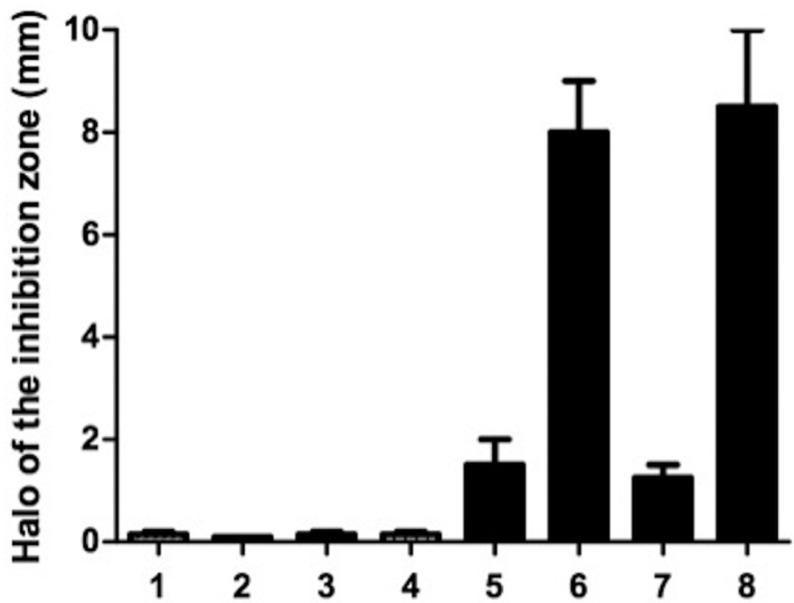
Antibacterial activity of the different pulp canal sealers evaluated by agar diffusion test. 5 mm in diameter and 2 mm deep disks composed of each pulp canal sealers were placed on agar plates previously incubated with incubated with *Enterococcus faecalis* and incubate at 37°C for 24h. All the assays were conducted in triplicate and the results were recorded in terms of the average diameter of inhibition zone (mm). Error bars indicate standard errors of the means. Statistically significant differences are indicated (Student’s t test; * *P* < 0.05; **P <0.01).

By using ADT test, the antibacterial effect is measured when a halo is formed, called inhibition zone, around the tested material on the agar plate. The size of this zone reflects the antibacterial effect of the sealer. Except for TotalFill BC Sealer, all the other sealers tested caused inhibition zones ( Table 2 and Fig. 1). BioRoot™RCS, MTA Fillapex and Sealapex Root Canal Sealer showed the lowest antibacterial activity compared to the others (bacterial inhibition zone; 0.2 ± 0.05, 0.3 ± 0.02 mm or 0.2 ± 0.04 respectively). Instead, a significant increase (*P*<0.05) in antibacterial effect for both EWT and AH plus sealers (bacterial inhibi-tion zone; 1.4 ± 0.3 or 1.2 ± 0.2 mm respectively) were found. Significantly higher (*P*<0.01) were the mean diameters of the bacterial inhibition zone by both EasySeal or N2 sealers (8.10 ± 0.2; 8.72 ± 0.4 mm, respectively). There was no bacterial growth on the control plates.

**Table 2:**
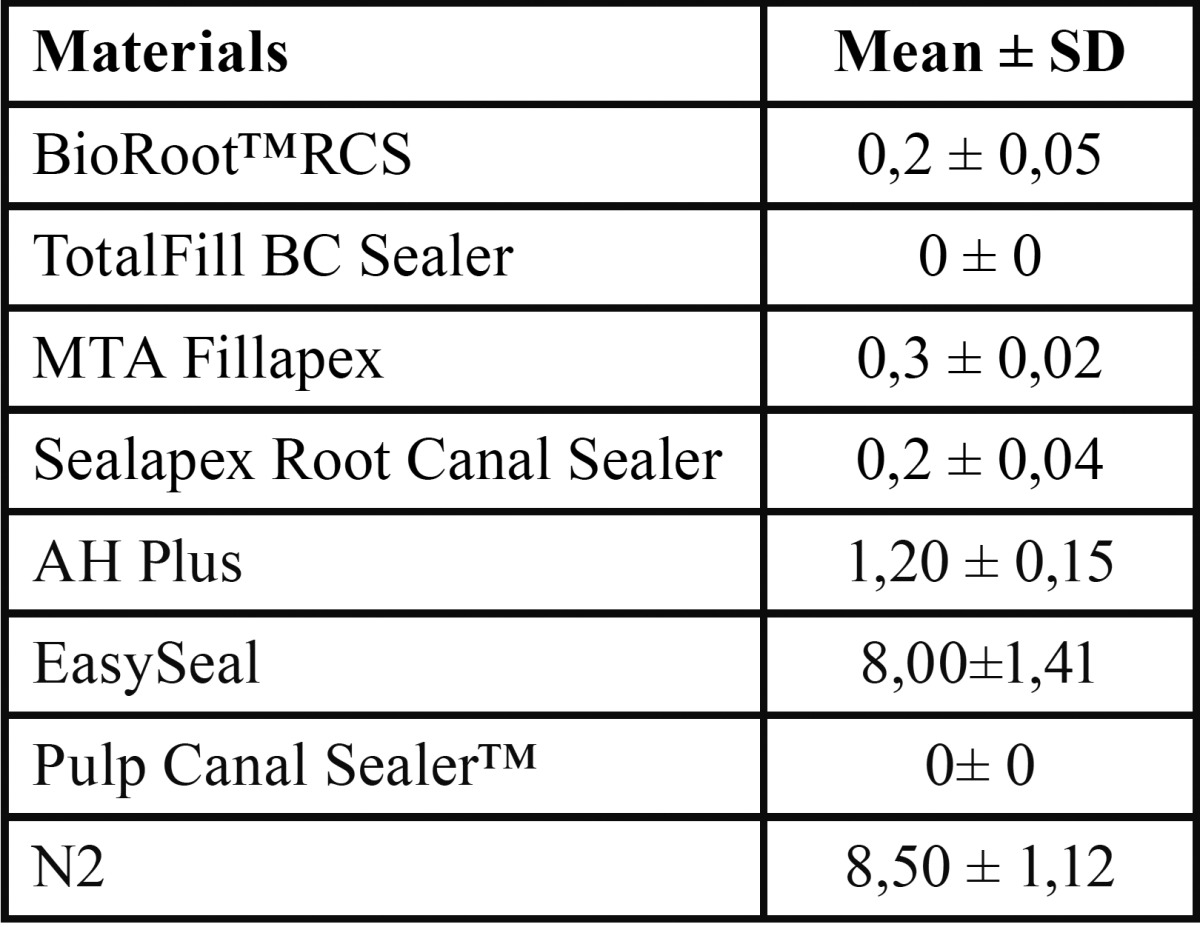
Mean diameter ± standard deviation (mm) of the bacterial inhibition zone by pulp canal sealers evaluated after 48h by ADT. 5 mm in diameter and 2 mm deep disks composed of each pulp canal sealers were placed on agar plates previously incubated with *Enterococcus faecalis* and incubate at 37°C for 24h. All the assays were conducted in triplicate and the results were recorded in terms of the average diameter of inhibition zone (mm).

Direct contact test (DCT)

**Figure 2:**
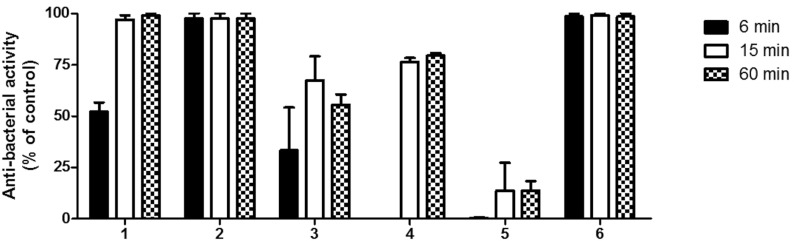
Antibacterial activity of the endodontic sealers at different experimental times on *Enterococcus faecalis* by direct contact test. Antibacterial activity is expressed as percentage of that observed in the absence of the sealer (0%). The data points are the means +/- SD of three independent experiments each performed in triplicate. Asterisk (*) indicates no statistically significant differences between the bacterial cells treated with sealer saline suspension or the sealer-free saline suspension (control).

DCT was used to evaluate the bactericidal effect of the sealers by directly calculate the exact numbers of surviving bacteria after each contact time (6, 15 and 60 min) by counting colony-forming units. Sealer-free saline suspension treated medium was considered as the negative control (set to 0% of antibacterial activity). The results of the test are shown in figure 2 and express as percentage of antibacterial activity compare to the negative control. All tested sealers were distinctly different from each other in their antimicrobial activity. AH plus and Sealapex Root Canal Sealer doesn’t show any bactericidal effect after 6 min of contact. After 15 and 60 min of contact a significant increment (*P*<0.05 for AH plus and *P*<0.01 for Sealapex Root Canal Sealer) of the bactericidal effect was found (Fig. 2). Significantly much higher was the antibacterial effect of Sealapex Root Canal Sealer com-pare to that observed for AH plus (*P*<0.01). BioRootTMRCS, MTA Fillapex, Pulp Canal Sealer™ and N2 showed at least means (4 ± 2 x 107/ml) of the number of colonies formed in milliliter after 6 min of contact. Except for N2, a significant increase in bactericidal effect (*P*<0.05) after 15 and 60 min for the other compared sealers (BioRootTMRCS, MTA Fillapex and Pulp Canal Sealer™). For every contact times considered, both TotalFill BC Sealer and EasySeal were bactericidal against *E. faecalis* and killed all bacteria.

## Discussion

Elimination of bacteria from root canal systems is crucial for the success of root canal treatment (10). Although many improvements have been achieved during recent years, the protocols of endodontic chemo-mechanical disinfection used today cannot predictably provide the sterility of root canal complex. As none of the elements of endodontic therapy (host defense system, instrumentation and irrigation, intracanal medicaments, permanent root ﬁlling and coronal restoration) can guarantee complete disinfection (11).

Numerous species of anaerobic bacteria such as *Streptococcus mutans* and *anginosus*, *Fusobacterium nucleatum*, *Staphylococcus aureus* were found in failed root canal therapy (12). However, *Enterococcus faecalis* is the microorganism which is usually related to the aetiology of persistent periradicular lesions (13). It possesses several virulence factors that contribute to its ability to survive the effects of conventional root canal therapy (14). Besides, this Gram-positive facultative anaerobe is able to invade dentine tubules and bind to collagen (15). For these reasons, *E. faecalis* was the microorganism selected in our research to evaluate the antimicrobial properties of the endodontic materials tested.

The endodontic sealers are used in root canal therapy to eliminate the microorganisms after the chemomechanical preparation and to prevent recolonization of the root canal system. It was recognized that following chemo-mechanical preparation of canals, the antimicrobial properties of endodontic sealers, could potentially control infections preventing the penetration of fluids into the root canal which can offer a nutrient supply to the remaining microorganisms. A sealer should be biocompatible and dimensionally stable, as well as having a long-lasting antibacterial effect (16).

The antibacterial effects of endodontic sealers have been investigated several times by using ADT and DCT (17). The ADT has been widely used to investigate the antimicrobial activity of sealers and it is one of the most common and simplex methods. However, it has some limitations such as lack of standardization of inoculum density, adequate culture medium, agar viscosity, plate-storage condition and dependency on the solubility and diffusion characteristic of both the test material and media (18). Thus, only water-soluble materials con be tested using ADT method (19). For this reason, ADT is no longer the only recommended test to evaluate the antibacterial activity of endodontic sealers. On the contrary, the DCT mimics the direct contact between microorganisms and the endodontic sealers inside a root canal and has several advantages such as reproducibility and quantitative assay (20). However, both methods have their own specific characteristic and it is difficult to compare their results.

In our study, the ADT results showed that the zinc-oxide based sealers have a higher antibacterial activity against *E. faecalis*. In particular, N2 showed the highest bactericidal effect; this finding can be attributed to the presence of paraformaldehyde in the composition of this sealer (21). EasySeal, which demonstrated an antimicrobial activity similar to N2, and EWT were ranked second and third respectively in term of inhibition halos. Similarly, to N2, these two materials belong to the family of zinc oxide based sealers and their antibacterial ability is probably caused by the existence of zinc oxide and thymoliodiden in their compositions. These results are comparable with the conclusions of other Authors on the efficacy of zinc-oxide based root canal sealers (22). AH plus reported a sizable antibacterial effect, similar to that demonstrated by EWT; its bactericidal activity may be explained by the presence of antibacterial components in epoxy resin (23). The results of ADT finally indicated no (or low) antibacterial activity for TotalFill BC Sealer, BioRoot™RCS, MTA Fillapex and Sealapex Root Canal Sealer. The reasons of this inability can be related to the absence of appropriate medium and permeation ability of these sealers. The results obtained by MTA Fillapex were in agreement with some studies (24), but in contrast with others (25).

In order to evaluate the antibacterial activity through DCT, the time of 60 minutes was suggested because in 6 and 15 minutes the sealers have no sufficient time to affect resisting bacteria such as *E. faecalis*. Owing to the dynamics of each sealer, the control group was considered separately through the course of the experiment. Hence, the study was conducted in three consecutive periods of 6, 15 and 60 minutes. In DCT sealers were placed in suspension; therefore, they were able to easily diffuse. Contrariwise in ADT, due to the bulky nature of the sealers, they were not able to easily diffuse and this can affect their antibacterial effects (26). For every contact times considered in DCT method, TotalFill BC Sealer and EasySeal were bactericidal against *E. faecalis* and killed all bacteria. These findings are in contrast with ADT, which demonstrated no antibacterial efficacy for TotalFill BC Sealer. Some studies have evaluated the cytocompatibility of these bio-ceramic sealers; however, to date no research has investigated their antibacterial effects. Probably, their longer setting time may be one of the factors that affected their permeability and therefore their results in ADT. This consideration is in contrast with the findings by Cobankara who found that time had no effect on antibacterial efficacy (27). The strong bactericidal ability of EasySael may be due by its high zinc-oxide content. Similarly, good percentage of antibacterial activity was found for EWT zinc-oxide based sealer. As regards the resinous sealers tested, AH plus and Sealapex doesn’t showed any bactericidal effect after 6 min; but after 15 and 60 min a significant increment in antibacterial activity was found, with a higher efficacy of Sealapex Root Canal Sealer. The bactericidal effect of these two sealers can be attributed to the antibacterial component in epoxy resin; however, the lowest long-time efficacy of AH Plus may be due by the paraformaldehyde released by this material only during setting period (28). Finally, N2 showed a fair antibacterial activity due to zinc-oxide and paraformaldehyde components; while MTA Fillapex showed a sizable bactericidal effect. This consideration is in contrast with a recent study by Morgental, which demonstrated a bactericidal effect of MTA Fillapex against *E. faecalis* before setting, but it did not maintain this ability 7 days after mixture (17).

## Conclusions

In the ADT N2 sealer showed maximum antibacterial activity and in the DCT TotalFill BC Sealer and EasySeal demonstrated the highest bactericidal effects. Therefore, the technique and components of the tested materials affected the antibacterial activity results.
